# The role of the unfolded protein response pathway in bone homeostasis and potential therapeutic target in cancer-associated bone disease

**DOI:** 10.1038/s41413-025-00457-6

**Published:** 2025-08-28

**Authors:** Molly E. Muehlebach, Sarah A. Holstein

**Affiliations:** 1 Cancer Research Doctoral Program, University of Nebraska Medical Center, Omaha, NE USA; 2 Department of Internal Medicine, University of Nebraska Medical Center, Omaha, NE USA

**Keywords:** Bone cancer, Bone

## Abstract

The unfolded protein response pathway is an evolutionarily conserved cytoprotective signaling cascade, essential for cell function and survival. Unfolded protein response signaling is tightly integrated with bone cell differentiation and function, and chronic unfolded protein response activation has been identified in bone disease. The unfolded protein response has been found to promote oncogenesis and drug resistance, raising the possibility that unfolded protein response modulators may have activity as anti-cancer agents. Cancer-associated bone disease remains a major cause of morbidity for patients with multiple myeloma or bone-metastatic disease. Understanding the critical role of unfolded protein response signaling in cancer development and metastasis, as well as its role in bone homeostasis, may lead to novel mechanisms by which to target cancer-associated bone disease. In this review, we summarize the current research delineating the roles of the unfolded protein response in bone biology and pathophysiology, and furthermore, review unfolded protein response modulating agents in the contexts of cancer and cancer-associated bone disease.

## Introduction

The endoplasmic reticulum (ER) is a distinct, membrane-bound organelle recognized for its role in protein synthesis, processing, and transport. As unfolded polypeptides enter the ER lumen, they undergo protein folding and disulfide bond formation via a multitude of enzymes and chaperone proteins.^1^ This ER microenvironment is specifically tailored for these processes, therefore physiological disturbances such as change in pH or lack of chaperone proteins, disrupts protein processing, increasing misfolded protein accumulation, ultimately inducing ER stress.^2,3^ To address this, the eukaryotic cell developed a cytoprotective signaling cascade termed the unfolded protein response (UPR) pathway.^4^ Upon sensing of ER stress, UPR signaling halts global protein translation while selectively promoting translation of ER chaperone proteins and removal processes such as ER-associated degradation (ERAD) and autophagy, to overcome misfolded protein loads.^5^ However, if UPR activation cannot restore proteostasis, the pro-apoptotic, terminal UPR signaling cascade is activated.

Due to the pro-survival role of the UPR, cancer cells commonly upregulate this signaling cascade in response to the hypoxic, nutrient deprived conditions of the tumor niche. This ultimately incurs a multitude of oncogenic benefits such as premetastatic niche formation, metastasis, and treatment resistance in the process.^6–8^ In fact, identification of elevated ER stress proteins in tumors corresponds with poor prognosis and adverse treatment outcomes in osteosarcoma (OS), multiple myeloma (MM), breast, prostate, and kidney cancer, all of which are cancers localized to (or which commonly metastasize to) the bone.^9–15^ The incidence of primary or metastatic bone cancer is relatively low (suspected to be about 5% of all cancer cases) with ~35 000 people in the United States dying each year with bone metastases.^16^ Whether bone-derived [OS, Ewing sarcoma (ES)] or bone-metastasizing, cancer cells drastically change the bone marrow microenvironment to promote their selective growth and survival, ultimately disrupting other essential processes occurring in the medullar cavity, such as bone remodeling.^17^

The homeostatic process of bone remodeling which occurs throughout adulthood is a tightly regulated process mediated by osteoclast bone-resorbing, osteoblast bone-forming, and osteocyte mechanosensory cells. Disruption of the remodeling cycle can result in bone loss or increased bone density, however in both cases the bone is brittle and can easily fracture. As a result, skeletal structure and integrity is compromised, significantly contributing to disease morbidity and negatively impacting overall patient survival in cancer-induced bone disease.^18,19^ The current treatment options for cancer-induced bone disease consist primarily of anti-osteoclastic agents such as nitrogen bisphosphonates (NBP) or tumor necrosis factor (TNF) superfamily member 11 (TNFSF11, also known as RANKL) monoclonal antibody denosumab.^20,21^ However, while these therapies show efficacy in reducing patient risk of further skeletal related events (SRE), whether they exhibit direct anti-neoplastic benefits has not been fully established.^20,22,23^ In recent years, chronic UPR activation has been identified in bone disease due to UPR signaling being tightly integrated with bone cell differentiation and function. Understanding the critical role of UPR signaling in cancer development and metastasis, as well as its role in bone homeostasis, suggests potential application for UPR-modulating therapeutics in cancer-associated bone disease. In this review, we summarize the current findings supporting the role of the UPR in bone biology and pathophysiology. Furthermore, we review UPR modulating agents, either FDA approved or currently undergoing clinical trial investigation, and discuss their dual anti-neoplastic and bone-related therapeutic potential.

## The UPR

**Fig. 1:**
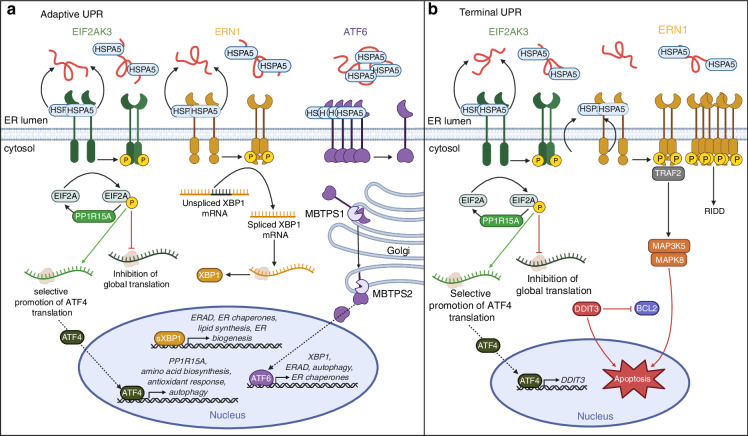
Signaling cascades of the UPR. **a** Adaptive UPR signaling consists of three major signaling cascades (EIF2AK3, ERN1, ATF6) and prioritizes absolution of ER stress in order to restore normal protein processing and cell function. EIF2AK3 is activated upon HSPA5 dissociation and binding of misfolded proteins in the ER lumen, promoting receptor dimerization and autophosphorylation. Activated EIF2AK3 phosphorylates EIF2A leading to global inhibition of protein translation with the exception of selective translation of ATF4 mRNA. ATF4 then mediates transcription of UPR target genes to promote amino acid biosynthesis, the antioxidant response, and autophagy to relieve ER stress. ATF4 also promotes transcription of phosphatase PP1R15A to dephosphorylate EIF2A and restore normal protein translation. HSPA5 dissociation from ERN1 leads to receptor dimerization and autophosphorylation. Activated ERN1 RNase splices XBP1 mRNA with translation generating transcriptionally active sXBP1. sXBP1 promotes transcription of genes associated with ERAD, lipid synthesis, ER biogenesis and ER chaperone expression in attempt to alleviate ER stress. Dissociation of HSPA5 from ATF6 allows for receptor translocation to the Golgi apparatus where it is proteolytically cleaved by MBTPS1 and MBTPS2 yielding its active transcriptional form. Transcriptionally active ATF6 then translocates to the nucleus where it promotes continuation of UPR signaling via induction of XBP1, ERAD, autophagy, and ER chaperone expression. **b** Terminal UPR, which is activated in response to chronic, prolonged ER stress. Persistent EIF2AK3-EIF2A translation of ATF4 activates expression of pro-apoptotic protein DDIT3 which inhibits BCL2, allowing for induction of apoptosis. Prolonged ERN1 activation can promote interactions with adaptor protein TRAF2 mediating downstream MAP3K5 and MAPK8-mediated apoptosis. Prolonged phosphorylation also favors higher order assembly of ERN1 oligomers, preventing XBP1 splicing and alternatively promoting regulated ERN1-dependent decay (RIDD). (Abbreviations: EIF2AK3 eukaryotic translation initiation factor 2 alpha kinase 3, ATF6 activating transcription factor 6, ERN1 ER to nucleus signaling 1, HSP70 HSPA5 heat shock protein family A member 5, EIF2A eukaryotic translation initiation factor 2 subunit alpha, p-EIF2A phosphorylated EIF2A, ATF4 activating transcription factor 4, XBP1 X-box-binding protein-1, sXBP1 spliced XBP1, MBTPS1 membrane bound transcription factor peptidase, site 1, MBTPS2 membrane bound transcription factor peptidase, site 2, DDIT3 DNA damage inducible transcript 3, Bcl-2 B-cell lymphoma 2, TRAF2 TNF receptor associate factor 2, MAP3K5 mitogen-activated protein kinase kinase kinase 5, MAPK8 mitogen-activated protein kinase 8)

The mammalian UPR consists of three, distinct signaling cascades, mediated by ER transmembrane proteins: eukaryotic translation initiation factor 2 alpha kinase 3 (EIF2AK3, also known as PERK), activating transcription factor 6 (ATF6), and ER to nucleus signaling 1 (ERN1, also known as IRE1) (Fig. 1a).^24^ Under basal conditions, ER chaperone, heat shock protein family (HSP70) member 5 (HSPA5, also known as GRP78 or BiP), occupies the luminal domains of EIF2AK3, ATF6, and ERN1, respectively, sterically hindering their activation. In the presence of misfolded proteins, HSPA5 dissociates allowing for receptor homodimerization and autophosphorylation [i.e., EIF2AK3, ERN1 or translocation to the Golgi (i.e., ATF6) for proteolytic processing]. EIF2AK3 activation initiates phosphorylation of eukaryotic translation initiation factor 2A (EIF2A), inhibiting global protein translation excluding activating transcription factor 4 (ATF4).^25^ ATF4 then activates downstream processes such as the antioxidant response, amino acid biosynthesis, and autophagy to ameliorate ER stress.^26^ ATF4 also induces expression of protein phosphatase 1 regulatory subunit 15A (PP1R15A, also known as GADD34) which promotes the dephosphorylation of phosphorylated EIF2A (p-EIF2A), attenuating ATF4 expression, and restoring protein synthesis.^27^ Alternatively, ERN1 activation mediates splicing of X-box binding protein 1 (XBP1) mRNA, yielding the active XBP1 splice variant (sXBP1). Active sXBP1 then translocates to the nucleus where it initiates transcription of genes involved in protein folding, secretion, and degradation.^28^ The ATF6 arm of the UPR is unique in that HSPA5 dissociation allows for translocation of ATF6 to the Golgi for proteolytic processing by both membrane bound transcription factor peptidase, site-1 (MBTPS1) and site-2 (MBTPS2). Transcriptionally active ATF6 then translocates to the nucleus where it stimulates expression of XBP1 along with other ER chaperones and reinforces ERAD and autophagic removal of misfolded proteins.^29^

These signaling cascades mediate the adaptive UPR, where pathway activation is focused on survival (Fig. 1a). However, when the adaptive signaling cascade cannot restore normal protein processing and folding, causing irremediable ER stress, terminal UPR signaling is activated (Fig. 1b). The terminal UPR is primarily mediated by ERN1 and EIF2AK3 signaling axes.^30^ Sustained EIF2AK3 activation leads to ATF4-mediated expression of pro-apoptotic transcription factor DNA damage-inducible transcript 3 (DDIT3, also known as CHOP) which inhibits anti-apoptotic protein B-cell lymphoma-2 (BCL2) for induction of BCL2 like 11 (BCL2L11) and apoptosis.^31^ Alternatively, prolonged ERN1 phosphorylation promotes higher order assembly of oligomers, attenuating XBP1 splicing, and alternatively favoring regulated ERN1-dependent decay (RIDD) for selective degradation of mRNA substrates.^32^ This leads to a downregulation in XBP1-mediated expression of ER chaperones, dampening the adaptive pro-survival axis. Furthermore, prolonged activation of ERN1 can promote assembly of adaptor protein TNF receptor associate factor 2 (TRAF2) with mitogen-activated protein kinase (MAPK) kinase kinase 5 (MAP3K5) activating downstream MAPK8-mediated apoptosis (Fig. 1b).^33,34^

## Genetic evidence for the UPR in skeletal disease

Chronic ER stress and resulting UPR-induced apoptosis is associated with a multitude of human diseases such as neurodegeneration, diabetes and cancer.^35–39^ Loss of UPR signaling components is associated with a variety of human skeletal dysplasias, therefore suggesting a functional role for UPR signaling in bone cell homeostasis, and insinuating a role for ER stress in bone disease.^4,40–43^ Of note, mutations in UPR proteins EIF2AK3, ATF4, and ATF6-like cAMP responsive element binding (CREB) transcription factors, have been shown to affect chondrocyte growth and overall bone health. However, due to this review highlighting the application of UPR treatment in adult bone remodeling, which does not directly involve chondrocytes, effects in chondrocytes will not be discussed. The following publications are recommended for more information on the UPR in chondrocytes.^40,44–46^

Wolcott-Rallison syndrome (WRS) is a rare, autosomal recessive disorder resulting from mutation in *EIF2AK3*. While the specific mutational event varies across families, these patients lack either partial or complete EIF2AK3 catalytic activity resulting in neonatal or early infancy type 1 diabetes, epiphyseal dysplasia, growth retardation, and osteoporosis.^47–49^ Liu et al. conducted a study analyzing how different single nucleotide polymorphisms (SNP) in the *EIF2AK3* gene impact bone mineral density (BMD) in two distinct populations. They discovered one nonsynonymous SNP to have borderline associations with decreased forearm BMD in both cohorts, and functional effects were revealed to be haplotype-specific with low-BMD associated with elevated levels of EIF2A, overall indicating a potential connection between BMD and EIF2AK3 functionality.

Coffin-Lowry Syndrome (CLS) is an X-lined condition characterized by severe intellectual disability, delayed development, craniofacial and skeletal abnormalities.^50,51^ CLS is caused by a mutation in *RPS6KA3*, encoding ribosomal S6 kinase A3, which is known to activate ATF4.^52^ In normal osteoblasts RPS6KA3 is thought to phosphorylate ATF4, inducing osteoblast specific gene expression and posttranslational regulation of type I collagen synthesis.^52^ Therefore, UPR dysfunction may contribute to the skeletal defects seen in CLS as theorized by lack of RPS6KA3 function.

Mutations in other UPR pathway components such as MBTPS1 and MBTPS2 are also associated with skeletal abnormalities. MBTPS1 and MBTPS2 mediate the cleavage and activation of various ER bound transcription factors like ATF6.^53^ An X-linked recessive form of osteogenesis imperfecta (OI), also referred to as brittle bone disease, has been attributed to defects in *MBTPS2*.^54^ Individuals with this mutation experience moderate to severe OI due to impaired MBTPS2-regulated intramembrane proteolysis of transcription factors ATF6, cAMP responsive element binding protein 3 like 1 (CREB3L1, also known as OASIS), and sterol regulatory element binding protein transcription factor 1 (SREBF1).^54^ Furthermore, multiple severe recessive forms of OI are associated with a mutations in *CREB3L1*, and ER stress inducible gene, cystine rich with EGF like domains 2 (*CRELD2*), is implicated in the pathogenesis of many skeletal dysplasias.^55,56^

Altogether, these genetic aberrations and resulting skeletal phenotypes suggest a critical role for UPR signaling in bone biology and begin to clarify its potential as a therapeutic target.

## The cycle of bone remodeling

Bone physiology and structure is constantly adapting throughout development with “bone modeling” (marrow formation and bone elongation) occurring during embryonic development, adolescence, and early adulthood, and “bone remodeling” (focal bone maintenance) occurring throughout the remainder of adulthood.^57^ The bone remodeling cycle consists of the characteristic “basic multicellular unit” (BMU) comprising osteoclasts, osteoblasts, osteocytes, and designated precursors.^58^ Various stimuli can activate the cycle of bone remodeling with the main initiators being mechanical stress, hormone secretion, and/or lack of bioavailable calcium or vitamin D. The cycle initiates when stimuli trigger expression of osteoclast differentiation factor TNFSF11 from osteocyte and/or osteoblast cells.^59,60^ TNFSF11 binds TNF receptor superfamily member 11a (TNFRSF11A, also known as RANK) on pre-osteoclast cells, promoting precursor migration and fusion.^61^ Osteoclast arrival and binding to the indicated bone site initiates sealing zone formation and acidification of the resorptive compartment.^62–64^ As old bone is resorbed, growth factors are released from the matrix which bind receptors on tissue-resident, mesenchymal type cells, referred to as skeletal stem cells (SSC), stimulating commitment to the osteoblast lineage.^65^ Fully differentiated osteoblasts then secrete new osteoid and promote matrix mineralization while dually signaling for osteoclast apoptosis.^66,67^ Once all osteoclasts have been removed and the osteoblasts have secreted sufficient new osteoid, Osteoblasts either embed themselves into the bone matrix becoming terminally differentiated osteocytes or become quiescent bone-lining cells (BLC), awaiting reactivation of the next bone remodeling cycle.^68^

The foundation for physiological bone maintenance is the controlled balance between bone resorption and bone formation, which starts with the number of mature osteoclast and osteoblast cells. Generation of these highly specialized cell types from their precursors requires a multitude of intracellular signaling cascades which have been elegantly described in a various publications.^69–75^ For the purpose of this review, we will briefly describe the main signaling cascades involved in osteoclast and osteoblast differentiation and function, which will later be re-examined with a UPR-integrated perspective.

## Osteoclast differentiation

**Fig. 2:**
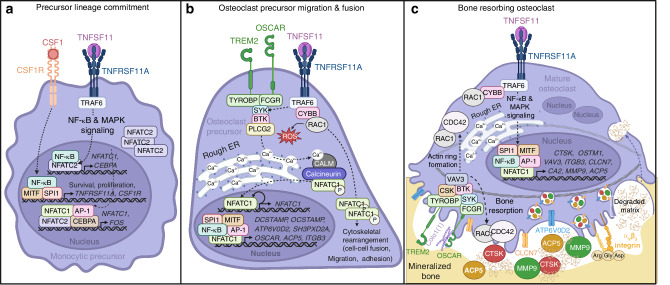
Signaling cascades during osteoclast differentiation. **a** CSF1 binds CSF1R on monocytic precursors activating transcription factors MITF and SPI1, promoting precursor survival and proliferation, and expression of TNFSF11 receptor (TNFRSF11A) and CSF1R. Surface expression of TNFRSF11A allows for TNFSF11 binding and activation of TRAF6 mediated NF-κB and MAPK signaling. Active NF-κB translocates to the nucleus where it promotes CEBPA expression and binds the NFATC1 promoter where it cooperates with NFATC2 to induce NFATC1 expression. CEBPA promotes expression of FOS which dimerizes with JUN to form active transcriptional complex AP-1. CEBPA and AP-1 in combination with NF-κB, NFATC2, and NFATC1 promote continued expression of FOS and NFATC1. **b** During TNFSF11-TNFRSF11A signaling, TRAF6 interacts with CYBB and RAC1 generating ROS and promoting cytoskeletal rearrangement necessary for precursor cell fusion, migration, and adhesion. TNFRSF11A signaling also mediates phosphorylation of adaptor proteins TYROBP and FCRG which activate downstream kinases, SYK, BTK, and PLCG2, triggering ER calcium release and activation of calmodulin (CALM)-dependent phosphatase calcineurin. Active calcineurin in turn dephosphorylates cytosolic NFATC1, allowing its translocation to the nucleus where it cooperates with transcription factors SPI1, MITF, AP-1, and NF-κB to promote osteoclast-specific gene expression. Additionally, NFATC1 binds its own promoter auto-amplifying its expression. **c** TNFRSF11A signaling continues to promote expression of proteins necessary for osteoclast resorption via NF-κB and MAPK signaling. α_V_β_3_ integrin or TREM2/OSCAR can bind RGD sequences on ECM proteins in the bone matrix initiating the resorptive signaling cascade mediated by TYROBP, CSK, SYK, RAC1, and CDC42 to regulate actin ring formation and bone resorption. Endosomal trafficking mediates acidification of the sealing zone via transport of proton pump ATP6V0D2 and chloride channel CLCN7 and release of proteases such as CTSK, MMP9, and ACP5. Degraded bone matrix is removed via transcytosis and released into the local microenvironment. (Abbreviations: CSF1 colony stimulating factor 1, CSF1R CSF1 receptor, TNFRSF11A tumor necrosis factor receptor family member 11A, TNFSF11 tumor necrosis factor soluble factor 11, MITF melanocyte-inducing transcription factor, TRAF6 tumor necrosis factor receptor-associated factor 6, MAPK mitogen-activated protein kinase, NFATC1 nuclear factor of activated T cells 1, CEBPA CCAAT enhancer binding protein alpha, AP-1 activator protein 1, TREM2 triggering receptor expressed on myeloid cells 2, OSCAR osteoclast-associated Ig-like receptor, TYROBP transmembrane immune signaling adaptor, FCRG Fc gamma receptor, SYK spleen associated tyrosine kinase, BTK Bruton’s tyrosine kinase, PLCG2 phospholipase C gamma 2, CALM calmodulin, CYBB cytochrome b-245 beta chain, ROS reactive oxygen species, DCSTAMP dendrocyte expressed seven transmembrane protein, OCSTAMP osteoclast stimulatory transmembrane protein, ATP6V0D2 ATPase H+ transporter V0 subunit d2, SH3PXD2A SH3 and PX domains 2A, ITGB3 integrin subunit beta 3, CLCN7 chloride voltage-gated channel 7, OSTM1 osteoclastogenesis associated transmembrane protein1, CTSK cathepsin K, ACP5 tartrate-resistant acid phosphatase 5, VAV3 vav guanine nucleotide exchange factor 3, CA2 carbonic anhydrase 2, MMP9 matrix metallopeptidase 9

Osteoclasts are derived from either erythromyeloid progenitors from the yolk sac (fetal osteoclasts—bone modeling) or circulating monocytes and bone marrow resident myeloid precursors (adult osteoclasts—bone remodeling).^76,77^ Differentiation is initiated upon binding of colony stimulating factor 1 (CSF1), produced primarily by MSCs and mature osteoblasts, to macrophage colony stimulating factor 1 receptor (CSF1R) on osteoclast precursors (Fig. 2a).^78,79^ Downstream CSF1 signaling promotes precursor survival and proliferation through activation of transcription factors Spi-1 proto-oncogene (SPI1, also known as PU.1) and melanocyte inducing transcription factor (MITF), in addition to surface expression of TNFRSF11A and CSF1R.^80,81^ Binding of TNFSF11, expressed either in its membrane-bound or free form, to TNFRSF11A on differentiating precursors recruits TNF receptor associated factor 6 (TRAF6) and mediates nuclear factor kappa B (NF-κB) and MAPK signaling.^79,82,83^ Active NF-κB localizes to the promoter of nuclear factor of activated T cells 1 (NFATC1) where it cooperates with transcription factor NFATC2 to induce NFATC1 expression.^84,85^ CCAAT/enhancer binding protein alpha (CEBPA), also thought to be a target of NF-κB signaling, promotes expression of essential osteoclast transcription factor, FOS.^86,87^ FOS forms a dimeric transcriptional complex with JUN family proteins creating activator protein 1 (AP-1) which cooperates with CEBPA to induce NFATC1 expression (Fig. 2a).^69,86^

As myeloid lineage cells, differentiating osteoclasts express immunoglobulin-like receptors, triggering receptor expressed on myeloid cells 2 (TREM2) and osteoclast-associated Ig-like receptor (OSCAR), with associated transmembrane immune signaling adaptor (TYROBP) and Fc gamma receptor (FCGR), respectively (Fig. 2b).^88^ TYROBP and FCRG contain immunoreceptor tyrosine-based activation motifs (ITAM) which mediate phosphorylation of spleen associated tyrosine kinase (SYK), Bruton’s tyrosine kinase (BTK), and phospholipase C gamma 2 (PLCG2) in cooperation with TNFRSF11A signaling.^89^ Phosphorylation of PLCG2 triggers intracellular calcium release, which then binds calcium-dependent kinase, calmodulin, which activates the downstream phosphatase calcineurin.^90^ Active calcineurin can then dephosphorylate cytosolic inactive NFATC1 therefore increasing levels of transcriptionally active protein.^90^ ER calcium release is also stimulated by cytochrome b-245 beta chain (CYBB, also known as NOX2) and cytosolic component RAC1, association with TRAF6.^91–93^ CYBB and Rac1 promote formation of reactive oxygen species (ROS) which stimulates ER calcium release, further promoting NFATC1 activation.^94^ NFATC1 cooperates with SPI1, MITF, and AP-1 to foster expression of osteoclast-specific genes to mediate precursor fusion (i.e., dendrocyte expressed seven transmembrane protein (*DCSTAMP*), osteoclast stimulatory transmembrane protein (*OCSTAMP*), ATPase H+ transporter V0 subunit d2 (*ATP6V0D2*), SH3 and PX domains 2A (*SH3PXD2A*), *OSCAR*, integrin subunit beta 3 (*ITGB3*) and bone resorption (i.e., chloride voltage-gated channel 7 (*CLCN7*), osteoclastogenesis associated transmembrane protein1 (*OSTM1*), cathepsin K (*CTSK*), tartrate-resistant acid phosphatase 5 (*ACP5*), vav guanine nucleotide exchange factor 3 (*VAV3)*, carbonic anhydrase 2 (CA2), and matrix metallopeptidase 9 (*MMP9*)) (Fig. 2b).^95–102^

Once all necessary structural and functional components are present, α_V_β_3_ integrin expressing osteoclasts can adhere to the bone surface initiating intracellular organization of podosome clusters and formation of the actin ring (Fig. 2c). Integrin engagement of Arg-Gly-Asp (RGD) sequences in ECM proteins (i.e., fibronectin, vitronectin, and osteopontin) activates the resorptive signaling cascade via TYROBP, nonreceptor tyrosine kinase SRC, SYK and downstream activation of RAC1 and CDC42.^103–106^ RAC1 and CDC42, Rho family small GTPases, regulate cytoskeletal rearrangement such as actin ring formation and apposition to the bone surface, in addition to facilitating resorptive processes at the ruffled boarder.^62^ Furthermore, transcytosis of degraded bone products mediates local release of growth factors, and endosomal trafficking of transporters and resorptive enzymes to the basolateral membrane facilitates sealing zone acidification and bone resorption (Fig. 2c).

## Osteoblast differentiation

**Fig. 3:**
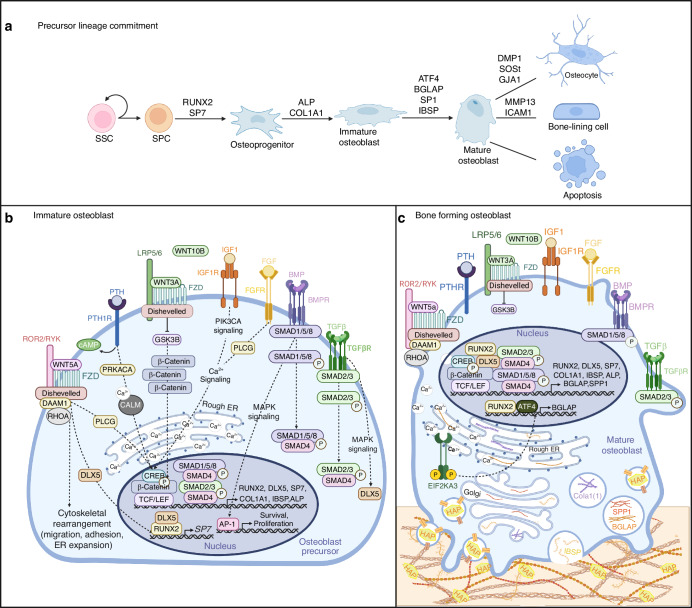
Signaling cascades during osteoblast differentiation. **a** Stepwise changes in gene expression throughout osteoblast lineage commitment and differentiation. **b** Multiple signaling cascades mediate the continued differentiation of immature osteoblast cells. TGFβ binds TGFβR and mediates RUNX2 and SP7 expression via induction of SMAD2/3 and SMAD4 transcriptional activation, and MAPK-mediated induction of DLX5. BMPs mediate precursor survival and proliferation via MAPK activation of AP-1 and promote osteoblast-specific gene expression via SMAD1/5/8 interactions with SMAD4. Canonical WNT signaling is activated by WNT3A or WNT10B binding FZD receptor allowing for binding to co-receptor LRP5/6. Binding inhibits GSK3B kinase activity, allowing for β-catenin to translocate to the nucleus where it interacts with TCF/LEF transcription factors to promote RUNX2 expression. Non-canonical WNT signaling is mediated by WNT5A binding FZD and interacting with co-receptor ROR2/RYK. Adaptor protein disheveled activates DAAM1 and RHOA mediating precursor migration, adhesion and ER expansion while also promoting DLX5-mediated induction of SP7 via MAPK signaling. PTH binding to PTH1R promotes production of cAMP, activating PRKACA, inducing calcium oscillation and activating transcription factor CREB to promote osteoblast-specific gene expression. Binding of growth factors IGF1 and FGF to cognate receptors activate PIK3CA signaling to induce PLCG2, further promoting calcium signaling and CREB mediated osteoblast gene expression. **c** Mature osteoblasts continue to promote osteoblast-specific gene expression via BMP, TGFβ, WNT, PTH, and growth factor signaling. RUNX2 and EIF2AK3 activation mediate induction of ATF4 which promotes expression of BGLAP. Mature osteoblasts begin to secrete matrix proteins such as BGLAP, SP1, IBSP, and COL1A1 to facilitate new bone formation, and secrete vesicles containing HAP to promote matrix maturation. (Abbreviations: SSC skeletal stem cell, SPC skeletal progenitor cell, RUNX2 runt-related transcription factor-2, SP7 Sp7 transcription factor, DMP1 dentin matrix acidic phosphoprotein 1, SOST sclerostin, GJA1 gap junction protein alpha 1, MMP13 matrix metallopeptidase 13, ICAM1 intracellular adhesion molecule 1, TGFβ transforming growth factor β, TGFβR TGFβ receptor, MAPK mitogen-activated protein kinase, DLX5 distal-less homeobox 5, BMP bone morphogenic protein, BMPR BMP receptor, AP-1 activator protein 1, LRP5/6 low-density lipoprotein receptor-related protein 5 or 6, FZD frizzled, TCF/LEF T cell factor/lymphoid enhancer factor, GSK3B glycogen synthase kinase 3 beta, FGF fibroblast growth factor, FGFR FGF receptor, IGF1 insulin-like growth factor 1, IGF1R IGF1 receptor, PIK3CA phosphatidylinositol-4,5-bisphosphate 3-kinase catalytic subunit alpha, PLCG phospholipase C gamma, PTH parathyroid hormone, PTH1R PTH1 receptor, PRKACA protein kinase cAMP-activated catalytic subunit alpha, CREB cAMP-response element binding protein, CALM calmodulin, ROR2/RYK receptor tyrosine kinase of the ROR-2 and Ryk families, DAAM1 disheveled associated activator of morphogenesis 1, EIF2AK3 eukaryotic initiation factor 2 alpha kinase 3, ATF4 activating transcription factor 4, BGLAP bone gamma-carboxyglutamate protein, SP1 secreted phosphoprotein 1, IBSP integrin binding sialoprotein, COL1A1 collagen type 1 alpha 1 chain, HAP hydroxyapatite)

Osteoblasts are derived from resident SSCs and multipotent progenitors (SSPC) which can differentiate into myoblasts, chrondrocytes, adipocytes, or osteoblasts.^107,108^ Early signaling events which favor osteoblast lineage commitment initiate the de-repression of runt-related transcription factor-2 (RUNX2) and downstream target osterix (SP7) (Fig. 3a).^109–114^ RUNX2^+^ and SP7^+^ osteoprogenitors continue to proliferate while promoting expression of collagen type 1 alpha chain 1 (COL1A1) and alkaline phosphatase (ALP) (Fig. 3a, b).^115^ At the end of this stage, immature osteoblasts are highly expressing ALP and COL1A1, and their proliferative potential is limited (Fig. 3b). RUNX2 induces expression of ATF4 and immature osteoblasts then begin to promote intracellular processing and secretion of bone matrix proteins, signaling the transition to osteoblast maturity (Fig. 3c).^52,115^ ATF4 interacts with RUNX2 and promotes expression of target genes bone gamma-carboxyglutamate protein (BGLAP, also known as osteocalcin) resulting in mature osteoblasts expressing secreted phosphoprotein 1 (SP1, also known as osteopontin), integrin binding sialoprotein (IBSP), ALP, and COL1A1. As osteoblasts lay down new osteoid, they dually mediate matrix maturation through secretion of matrix vesicles containing various transporters and enzymes that promote utilization of phosphate and calcium for matrix mineralization (Fig. 3c).^116^ From this point, terminally differentiated osteoblasts can either undergo apoptosis, become BLCs, or embed themselves into the bone matrix as terminally differentiated osteocytes. The specific molecular pathways which mediate the osteoblast to osteocyte or BLC transition are less understood, however elevated expression of dentin matrix acidic phosphoprotein 1, sclerostin, and gap junction protein alpha 1 (GJA1, also known as connexin 43) is characteristic of osteocytes while intercellular adhesion molecule 1 (ICAM1) and matrix metallopeptidase 13 (MMP13) expression is specific to BLCs (Fig. 3a).^117–119^

Other growth factors and cytokines, either circulatory or from the local microenvironment, further promote RUNX2, SP7, and other osteoblast-specific genes to promote differentiation (Fig. 3b, c). Fibroblast growth factor (FGF) and insulin like growth factor 1 (IGF1) bind receptor tyrosine kinases on osteoblast precursors and initiate MAPK, phosphatidylinositol-4,5-bisphosphate 3-kinase catalytic subunit alpha (PIK3CA), and PLCG2 signaling cascades to promote proliferation, survival, and continued RUNX2 expression.^120,121^ Bone morphogenic proteins 2, 4, 7 (BMP) or transforming growth factor β (TGFβ), bind cognate receptors on osteoblast precursors and initiate SMAD1/5/8 or SMAD2/3 signaling respectively, which cooperate with SMAD4 and promote expression of RUNX2 and SP7.^109,122^ BMP signaling also regulates osteogenesis through a SMAD-independent mechanism, in which transcription factor, distal-less homeobox 5 (DLX5) mediates RUNX2 and SP7 expression through MAPK signaling.^123,124^ Parathyroid hormone (PTH), known to exhibit anabolic effects on the skeleton, binds PTH-1 receptor (PTH1R) on pre-osteoblasts activating heterotrimeric G protein stimulation, production of cAMP, induction of protein kinase cAMP-activated catalytic subunit alpha (PRKACA), calcium oscillation, and activating transcription factor cAMP-response element binding protein (CREB) to promote osteoblast-specific gene expression.^125^ Interestingly, PTH binding to PTH1R can form a signaling complex with WNT pathway protein low-density lipoprotein receptor related protein 5 or 6 (LRP5/6), activating canonical WNT signaling in the absence of a WNT ligand.^126,127^ Alternatively, canonical WNT signaling is activated primarily by ligands WNT3A or WNT10B with noncanonical signaling induced by WNT5A.^128,129^ Canonical signaling follows WNT binding receptor frizzled (FZD) and co-receptor LRP5/6 on pre-osteoblasts, promoting β-catenin translocation to the nucleus where interactions with transcription factors T cell factor/lymphoid enhancer factor (TCF/LEF) increase RUNX2 levels to inhibit adipogenesis (i.e., CEBPA, peroxisome proliferator activated receptor gamma (PPARG)).^126,130–132^ Alternatively, non-canonical WNT signaling promotes calcium oscillation to induce SP7 expression, in addition to activating RHOA for cytoskeletal rearrangement and MAPK8 signaling to promote DLX5 expression (Fig. 3b, c).^133,134^

## Osteoblasts and the UPR

Osteoblasts rely on intracellular secretory processes to facilitate collagen production and secretion. As a result, differentiating osteoblasts require ER expansion and upregulation of UPR proteins to proactively mitigate proteotoxic stress. Skeletal dysplasias such as osteogenesis imperfecta (OI), characterized by mutations in the type I collagen gene, display abnormal osteoblasts possessing misfolded collagen proteins indicative of chronic ER stress. Due to accumulation of mutated collagen and inability to restore normal collagen folding, ER stress may mediate osteoblast apoptosis seen in OI and other osteoblast-related pathologies.^135^

The critical role of EIF2AK3 in osteoblast biology was confirmed by global knockout of EIF2AK3 (*EIF2AK3*^*−/−*^*)* in mice resulting in a similar phenotype to that in WRS, such as growth retardation and severe osteopenia.^136–139^ Specifically, osteoblast defects were determined to result from impaired collagen processing and secretion, and decreased mineralization capacity.^137^ Expression of genes indicating osteoblast maturity such as ALP, COL1A1, BGLAP, and IBSP were also found to be reduced in the skeletal tissue of *EIF2AK3*^*−/−*^ mice.^136^ This could be due to loss of PTH-mediated ATF4 expression and promotion of osteogenesis. Systemic knockout of EIF2AK3 downstream target *ATF4*^*−/−*^ or its regulatory kinase, *RPS6KA3*^*−/−*^, results in severe osteopenia and phenotypes similar to WRS and CLS.^52,139^ With phenotypes of *ATF4*^*−/−*^*, RPS6KA3*^*−/−*^, and *EIF2AK3* ^*−/−*^ mice suggesting a functional role of EIF2AK3/ATF4 signaling in bone development, it is important to note that secondary effects on bone due to gene inactivation in non-skeletal tissues cannot be excluded. Evidence that ATF4 is at least partially dispensable for early SSPC differentiation led to the discovery that ATF4 and its downstream target RPS6KA3, post-transcriptionally mediate COLα1(1) synthesis and expression of IBSP and BGLAP, indicating a critical role in late-stage osteoblast differentiation and function.^140^ This is supported by in vitro data utilizing the small molecule salubrinal, which inhibits the dephosphorylation of EIF2A, allowing for prolonged EIF2A inhibition of protein translation and selective ATF4 expression. Studies looking at the effects of salubrinal treatment on MC3T3-E1 osteoblast-like cells showed increased ATF4 expression and mineralization capacity, indicating a potential bone-forming benefit.^141^ Further investigation revealed PTH induces ER stress activating HSP90-dependent EIF2AK3/EIF2A-mediated ATF4 expression.^142,143^ Furthermore, salubrinal was found to mediate osteoblast expression of autophagy marker ATG7, alleviating ER stress.^144^ Additional in vitro studies utilizing pro-osteogenic cytokine BMP2 found BMP2 induced mild ER stress and EIF2AK3-mediated ATF4 activation, ultimately regulating expression of osteoblast genes BGLAP and IBSP.^139^

Tohmonda et al. have extensively studied the role of ERN1/XBP1 signaling in osteoblast biology by utilizing immortalized mouse embryonic fibroblasts (mEF). Due to global ERN1 knockout being embryonically lethal, mEFs derived from *ERN1*^*−/−*^ embryos were used.^145^ Utilizing BMP-2 to stimulate mEF differentiation, they found *ERN1*^*−/−*^ mEFs failed to mature into osteoblasts, also seen with ERN1 and XBP1-siRNA-transfected mEFs.^145^ Through stimulation of ER stress, they found mEFs and MC3T3-E1 cells to induce SP7 expression in an XBP1-dependent and TRAF2-independent manner.^145^ Further investigation into ERN1/XBP1 pathway targets revealed BMP2 induced XBP1 cleavage which upregulated PTH1R expression on the osteoblast cell surface.^146^ However, this could indicate an indirect effect on osteoclast differentiation with PTH binding of PTH1R inducing osteoblast TNFSF11 expression. This was confirmed by findings from Iyer et al. which found that the ER stress inducer tunicamycin induced TNFSF11 expression in primary osteoblast cultures. Tunicamycin inhibits N-linked glycosylation in the ER, resulting in misfolded proteins and activation of all 3 UPR pathways in osteoblast cells via increased levels of p-EIF2A cleaved ATF6, and sXBP1.^147^ Administration of tunicamycin to adult mice caused ER enlargement in osteoblasts and osteocytes, indicating ER stress, as well as increased TNFSF11 expression and osteoclast number.^147^ However overall changes in bone mass were not reported.^147^ These studies suggest aberrant ER stress can promote a vicious osteolytic cycle through UPR-mediated induction of osteoblast TNFSF11 expression.

**Fig. 4:**
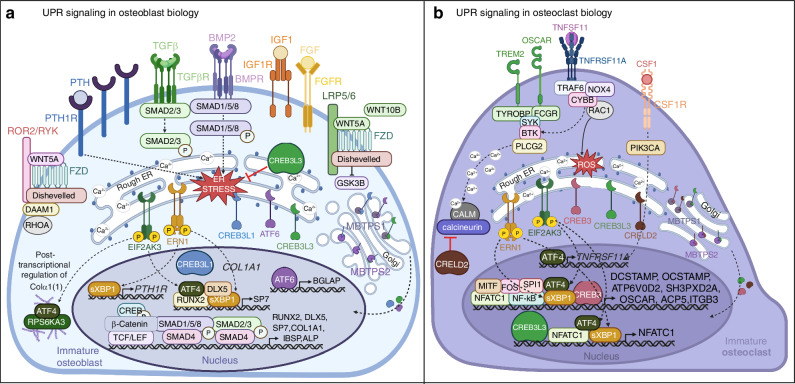
Integration of UPR signaling cascades in bone cell differentiation. **a** BMP-2 SMAD1/5/8 signaling and non-canonical WNT signaling promote ER stress and UPR pathway activation. EIF2AK3 activation induces ATF4 which promotes expression of SP7 and interacts with RPS6KA3 to post-transcriptionally regulate COLα1(1) processing. ERN1 splices XBP1 with sXBP1 promoting expression of SP7 and PTH1R. Activated ATF6 and CREB3L1 translocate to the Golgi where they are proteolytically processed to produce their active transcriptional form. In the nucleus, ATF6 promotes BGLAP expression and CREB3L1 promotes COL1A1 expression. CREB3L3 is also activated upon ER stress with its active transcriptional form negatively regulating osteoblast signaling. **b** TNFRSF11A-TNFSF11 signaling stimulates calcium signaling and ROS production which induce ER stress and UPR activation. ERN1 and EIF2AK3 activation mediates ATF4 and sXBP1 promotion of NFATC1 and other osteoclast-specific genes. ATF4 also mediates CSF1 induced surface expression of TNFRSF11A. Activated CREB3, CREB3L3, and CRELD2 translocate to the Golgi where they are proteolytically processed to produce their active transcriptional form. In the nucleus, CREB3L3 promotes NFATC1 expression and CREB3 promotes expression of other osteoclast-specific genes. Alternatively, CRELD2 negatively regulates osteoclast differentiation via inhibition of ER calcium release. (Abbreviations: EIF2AK3 eukaryotic initiation factor 2 alpha kinase 3, ERN1 ER to nucleus signaling 1, XBP1 X-box-binding protein-1, sXBP1 spliced XBP1, MBTPS1 membrane bound transcription factor peptidase, site 1, MBTPS2 membrane bound transcription factor peptidase, site 2, RUNX2 runt-related transcription factor-2, SP7 Sp7 transcription factor, TGFβ transforming growth factor β, TGFβR TGFβ receptor, MAPK mitogen-activated protein kinase, DLX5 distal-less homeobox 5, BMP bone morphogenic protein, BMPR BMP receptor, AP-1 activator protein 1, LRP5/6 low-density lipoprotein receptor-related protein 5 or 6, FZD frizzled, TCF/LEF T cell factor/lymphoid enhancer factor, GSK3B glycogen synthase kinase 3 beta, FGF fibroblast growth factor, FGFR FGF receptor, IGF1 insulin-like growth factor 1, IGF1R IGF1 receptor, PIK3CA phosphatidylinositol-4,5-bisphosphate 3-kinase catalytic subunit alpha, PLCG phospholipase C gamma, PTH parathyroid hormone, PTH1R PTH1 receptor, PRKACA protein kinase cAMP-activated catalytic subunit alpha, CREB cAMP-response element binding protein, CALM calmodulin, ROR2/RYK receptor tyrosine kinase of the ROR-2 and Ryk families, DAAM1 disheveled associated activator of morphogenesis 1, ATF4 activating transcription factor 4, BGLAP bone gamma-carboxyglutamate protein, SP1 secreted phosphoprotein 1, IBSP integrin binding sialoprotein, COL1A1 collagen type 1 alpha 1 chain, RPS6KA3 ribosomal S6 kinase A3, CREB3L1 cAMP responsive element binding protein 3 like 1, CREB3L3 cAMP responsive element binding protein 3 like 3, CSF1 colony stimulating factor 1, CSF1R CSF1 receptor, TNFRSF11A tumor necrosis factor receptor family member 11A, TNFSF11 tumor necrosis factor soluble factor 11, MITF melanocyte-induced transcription factor, TRAF6 tumor necrosis factor receptor-associated factor 6, MAPK mitogen-activated protein kinase, NFATC1 nuclear factor of activated T cells 1, CEBPA CCAAT enhancer binding protein alpha, AP-1 activator protein 1, TREM2 triggering receptor expressed on myeloid cells 2, OSCAR osteoclast-associated Ig-like receptor, TYROBP transmembrane immune signaling adaptor, FCRG Fc gamma receptor, SYK spleen associated tyrosine kinase, BTK Bruton’s tyrosine kinase, PLCG2 phospholipase C gamma 2, CALM calmodulin, CYBB cytochrome b-245 beta chain, ROS reactive oxygen species, DCSTAMP dendrocyte expressed seven transmembrane protein, OCSTAMP osteoclast stimulatory transmembrane protein, ATP6V0D2 ATPase H+ transporter V0 subunit d2, SH3PXD2A SH3 and PX domains 2A, ITGB3 integrin subunit beta 3, CLCN7 chloride voltage-gated channel 7, OSTM1 osteoclastogenesis associated transmembrane protein1, CTSK cathepsin K, ACP5 tartrate-resistant acid phosphatase 5, VAV3 vav guanine nucleotide exchange factor 3, CA2 carbonic anhydrase 2, MMP9 matrix metallopeptidase 9, CREB3 cAMP responsive element binding protein 3, CRELD2 cystine rich with EGF like domains 2

*ATF6*^−/−^ mice with systemic inactivation of the protein, demonstrate no apparent defects, but the mice are more sensitive to chemically-induced ER stress compared to wild-type.^29^ Interestingly, ATF6 also contains the osteoblast-specific cis-acting element 2 (OSE2) motif, and through various loss and gain of function studies utilizing MC3T3-E1 cells, Jang et al. found ATF6 positively regulates BGLAP expression and matrix mineralization.^148^ However, *ATF6*^*−/−*^ mice lacked an overt phenotype suggesting ATF6 as dispensable for bone development and maintenance.^29^ Global knockout of structurally homologous protein, *CREB3L1*^*−/−*^, resulted in osteoporotic mice exhibiting a similar phenotype to CREB3L1-deficiency in humans.^149,150^ Selective reintroduction of *CREB3L1* into osteoblast cells of *CREB3L1*^*−/−*^ mice restored bone density suggesting a direct regulatory role of CREB3L1 in osteoblast function.^150^ Importantly, osteoblasts of *CREB3L1*^*−/−*^ mice exhibited enlarged rough ERs with accumulation of bone matrix proteins such as BGLAP and procollagen.^149,150^ Reintroduction of CREB3L1 rescued ER morphology suggesting CREB3L1 modulates osteoblast intracellular processing and secretion of bone matrix proteins.^150^ Furthermore, CREB3L1 was found to bind the osteoblast-specific COL1A1 promoter via a UPR element (UPRE)-like sequence, implicating CREB3L1 as a mediator of UPR-mediated COL1A1 expression and processing in osteoblast cells.^149^ Interestingly, cAMP response element binding protein 3 like 3 (CREB3L3), also structurally homologous to ATF6, negatively regulates BMP2-mediated osteogenic signaling, suggesting a role in preventing overactivation of osteogenesis (Fig. 4a).^151^

## Osteoclasts and the UPR

Having determined that ERN1/XBP1 signaling influences osteoblast biology, Tohmonda et al. confirmed an additional impact on osteoclast biology. UPR activation was found to occur during early osteoclastogenesis with TNFSF11-treated bone marrow-derived macrophages (BMM) exhibiting a transient increase in spliced *XBP1* transcripts.^152^ This was confirmed to result from ERN1/XBP1 signaling and found it to be essential for osteoclastogenesis in vitro with siRNA silencing of *ERN1* in wild-type (WT) BMMs found to diminish differentiation and resorption. To evaluate the role of ERN1 in vivo they generated *ERN1*^*flox/flox*^*/Mx1-Cre* mice (*ERN1*^*Mx1*^) which depleted ERN1 from the BMM cells. *ERN1*^*Mx1*^ mice exhibited significantly increased bone mass and decreased osteoclast numbers compared to WT.^152^ To confirm these effects were due to defective osteoclastogenesis, *ERN1* transcript levels in *ERN1*^*Mx1*^ osteoblasts were evaluated and while a moderate reduction (~27%) was confirmed, no significant changes in osteoblast marker expression were evident. To further validate that decreased bone mass results from osteoclasts lacking ERN1, lethally irradiated WT mice were injected with *ERN1*^*Mx1*^ BMMs and maintained for 12 weeks. Mice reconstituted with *ERN1*^*Mx1*^ BMs exhibited a higher bone mass with decreased osteoclast number and resorption compared to mice reconstituted with WT BMMs.^152^ With luciferase reporter assays confirming potential XBP1 binding sites in the *NFATC1* promoter, decreased osteoclastogenesis in vivo may be the result of decreased *NFATC1* expression. To make the final connection between the UPR and osteoclastogenesis they determined TNFSF11-TNFRSF11A signaling to induce transient ER stress partially through oscillation of intracellular calcium levels promoting ERN1 endonuclease activity, XBP1 splicing, and *NFATC1* expression.^152^

As previously mentioned, Wei et al. found global loss of EIF2AK3 significantly decreased osteoblast number and functional capacity, but effects on osteoclast number and functionality was not reported.^136^ However, it was reported that ACP5 and CTSK expression in the long bones of *EIF2AK3* ^*−/−*^ mice was depleted by 55% compared to WT.^136^ ACP5 enzyme activity in the serum and bone extracts was also reduced and total bone protein levels of CTSK were depleted.^136^ Interestingly, impaired osteoclast activity in *EIF2AK3* ^*−/−*^ mice was partially correlated to reduced bone TNFSF11 expression. In aggregate these studies suggest that overall bone loss in EIF2AK3^−/−^ mice is due to reduction in bone formation and depletion of TNFSF11-expressing osteoblasts, suggesting reduced osteoblast-mediated activation of osteoclast differentiation and resorption. Therefore, whether reduced resorption was a direct result of EIF2AK3 loss in osteoclast cells is unclear. A direct role was confirmed with use of EIF2AK3 inhibitor GSK2606414 in vitro which revealed significant inhibitory effects on osteoclast differentiation and resorption through disruption of TNFSF11-induced MAPK and NF-κB signaling.^153^ Additionally, evidence of EIF2AK3 activation in fluoride-induced bone resorption suggests a potential role for EIF2AK3 in osteoclast activation.^154^

Cao et al. set out to establish whether ATF4 played a direct role in osteoclast biology utilizing global knockout *ATF4* (*ATF4*^*−/−*^) mice.^155^ Initial evaluations found *ATF4*^*−/−*^ mice exhibited decreased osteoclast number and ACP5 activity in tibiae compared to WT. Furthermore, analysis of BMMs from *ATF4*^*−/−*^ mice in vitro revealed a dramatic reduction in differentiation potential and bone resorption. Utilizing the *ACP5* promoter they generated an osteoclast-targeted transgenic mouse model where they overexpressed ATF4 (*ACP5-ATF4-tg*). *ACP5-ATF4-tg* mice displayed a severe osteopenic phenotype with a significant reduction in bone mass and trabecular number. Furthermore, evidence of significantly increased ACP5 activity and osteoclast number in *ACP5-ATF4-tg* tibiae compared to control confirmed that ATF4 overexpression increased osteoclast differentiation and resorption in vivo.^155^ Ultimately, ATF4 was determined to be a critical downstream target of CSF1-PIK3CA signaling during early osteoclast differentiation, and found to be required for CSF1 induction of TNFRSF11A expression in BMMs.^155^ Furthermore, ATF4 was associated with NFATC1 in a TNFSF11-dependent manner.^155^ However, in vitro studies looking at the effects of salubrinal, therefore prolonging ATF4 expression, on TNFSF11-stimulated osteoclastogenesis revealed an inhibitory effect on NFATC1 expression and RAC1 GTPase activity, suggesting a potential negative feedback loop mediated by ATF4.^156–160^ This inhibitory effect on osteoclastogenesis could explain why salubrinal treatment was found to ameliorate bone loss in models of OVX-induced, TNFSF11-induced, and disuse osteoporosis and even accelerated bone healing after injury in vivo.^141,160–162^

The role of ATF6 in osteoclastogenesis is less well known but seems to be mostly unaffected by UPR-induced osteoclastogenesis.^163^ Structurally homologous ER bound transcription factors CREB3L3, CREB3L1, and cAMP response element-binding protein 3 (CREB3 or LUMAN) have been suggested to play a role in UPR-mediated bone effects.^164,165^ CREB3 expression was found to be induced during osteoclastogenesis directly stimulating cell fusion through induction of DCSTAMP.^166^ However, CREB3 activation was determined ultimately independent of ER stress signaling in that CREB3 was only detectable upon TNFSF11 stimulation and not upon induction of physiological ER stress.^166^ Further investigation emphasized the role of calcium signaling and generation of reactive oxygen species (ROS) during osteoclast differentiation which may mediate ER stress and UPR activation. For example, ROS production and calcium flux during osteoclast differentiation were found to induce ERN1-mediated promotion of osteoclast markers, ACP5 and CTSK.^167–169^ Similarly, TNFSF11-induced ROS generation was found to activate CREB3L3 promotion of NFATC1 expression, with inhibition of ROS production using *N*-acetylcysteine (NAC) significantly reducing TNFSF11-induced CREB3L3 activation and NFATC1 expression.^170^ Furthermore, NADPH oxidase 4 (NOX4), known to mediate TNFSF11-induced autophagy and osteoclastogenesis, was found to activate EIF2AK3/EIF2A/ATF4 signaling.^171^ EIF2AK3 signaling activation was determined to result from elevated levels of non-mitochondrial ROS from TNFSF11-mediated induction of autophagy.^171^ Use of the selective ER calcium-dependent ATPase inhibitor thapsigargin, which depletes intracellular calcium stores activating UPR signaling, revealed thapsigargin to have a biphasic effect on osteoclastogenesis with mild stress levels promoting osteoclast signaling and drastic, prolonged calcium depletion leading to caspase-3 cleavage and apoptosis.^168^ Thapsigargin has been shown to upregulate EIF2A, EIF2AK3, HSPA5, and ERN1 during osteoclast differentiation promoting TNFSF11-induced NF-κB activity.^153,163,172^ Alternatively, CRELD2, was found to negatively regulate osteoclast differentiation through inhibition of ER calcium release and impaired calcium-dependent activation of NFATC1 (Fig. 4b).^55^

## UPR-modulators and potential applications in cancer-associated bone disease

**Fig. 5:**
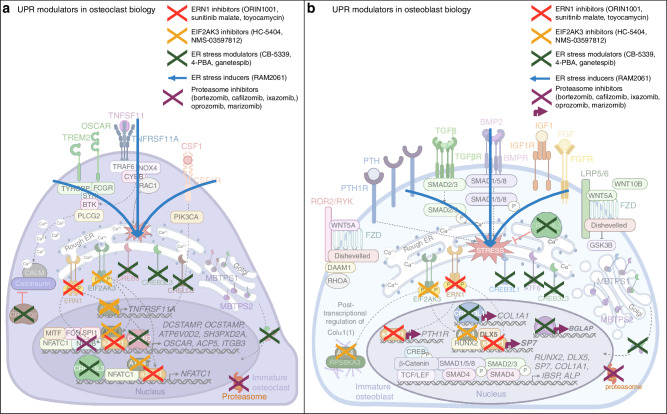
Potential effects of UPR modulating agents on bone cell differentiation. **a** Inhibition of UPR signaling with ERN1 inhibitors, EIF2AK3 inhibitors, or ER stress modulators disrupts osteoclast-specific gene expression mediated by ATF4, sXBP1, CREB3, and CREB3L3. Alternatively, ER stress inducers may promote osteoclast differentiation through induction of the UPR and induction of osteoclast genes. Proteasome inhibitors disrupt proteasomal degradation preventing NF-κB-mediated signaling and gene expression. **b** Inhibition of UPR signaling with ERN1 inhibitors, EIF2AK3 inhibitors, or ER stress modulators disrupts osteoblast-specific gene expression mediated by ATF4, sXBP1, ATF6, and CREB3L1. ER stress inducers may promote osteoblast differentiation through induction of the UPR and induction of osteoblast genes. Proteasome inhibitors disrupt proteasomal degradation inducing the UPR and promoting osteoblast-specific gene expression by ATF4, sXBP1, ATF6, and CREB3L1 and ATF4 mediated COLα1(1) processing. (Abbreviations: EIF2AK3 eukaryotic initiation factor 2 alpha kinase 3, ERN1 ER to nucleus signaling 1, XBP1 X-box-binding protein-1, sXBP1 spliced XBP1, MBTPS1 membrane bound transcription factor peptidase, site 1, MBTPS2 membrane bound transcription factor peptidase, site 2, CSF1 colony stimulating factor 1, CSF1R CSF1 receptor, TNFRSF11A tumor necrosis factor receptor family member 11A, TNFSF11 tumor necrosis factor soluble factor 11, MITF melanocyte-induced transcription factor, TRAF6 tumor necrosis factor receptor-associated factor 6, MAPK mitogen-activated protein kinase, NFATC1 nuclear factor of activated T cells 1, AP-1 activator protein 1, TREM2 triggering receptor expressed on myeloid cells 2, OSCAR osteoclast-associated Ig-like receptor, TYROBP transmembrane immune signaling adaptor, FCRG Fc gamma receptor, SYK spleen associated tyrosine kinase, BTK Bruton’s tyrosine kinase, PLCG2 phospholipase C gamma 2, ACP5 tartrate resistant acid phosphatase 5, CREB3 cAMP responsive element binding protein 3, CRELD2 cystine rich with EGF like domains 2, DCSTAMP dendrocyte expressed seven transmembrane protein, OCSTAMP osteoclast stimulatory transmembrane protein, ATP6V0D2 ATPase H+ transporter V0 subunit d2, SH3PXD2A SH3 and PX domains 2A, ITGB3 integrin subunit beta 3, ACP5 tartrate-resistant acid phosphatase 5, RUNX2 runt-related transcription factor-2, SP7 Sp7 transcription factor, TGFβ transforming growth factor β, TGFβR TGFβ receptor, MAPK mitogen-activated protein kinase, DLX5 distal-less homeobox 5, BMP bone morphogenic protein, BMPR BMP receptor, AP-1 activator protein 1, LRP5/6 low-density lipoprotein receptor-related protein 5 or 6, FZD frizzled, TCF/LEF T cell factor/lymphoid enhancer factor, GSK3B glycogen synthase kinase 3 beta, FGF fibroblast growth factor, FGFR FGF receptor, IGF1 insulin-like growth factor 1, IGF1R IGF1 receptor, PLCG phospholipase C gamma, PTH parathyroid hormone, PTH1R PTH1 receptor, CREB cAMP-response element binding protein, ROR2/RYK receptor tyrosine kinase of the ROR-2 and Ryk families, DAAM1 disheveled associated activator of morphogenesis 1, ATF4 eukaryotic activating transcription factor 4, BGLAP bone gamma-carboxyglutamate protein, SP1 secreted phosphoprotein 1, IBSP integrin binding sialoprotein, COL1A1 collagen type 1 alpha 1 chain, RPS6KA3 ribosomal S6 kinase A3, CREB3L1 cAMP responsive element binding protein 3 like 1, CREB3L3 cAMP responsive element binding protein 3 like 3)

While UPR modulators have demonstrated potential bone related benefits in vivo, translation of these agents to clinical use in bone disease has yet to be achieved. Current treatment options for cancer-induced bone disease consist primarily of anti-osteoclastic agents such as NBPs or the monoclonal antibody denosumab. However, while these therapies show efficacy in reducing patient risk of further SRE, whether they exhibit an additional anti-neoplastic benefit has not been confirmed. Understanding the critical role of UPR signaling in cancer development and metastasis, as well as its role in bone homeostasis, suggests potential application for UPR-modulating therapeutics in cancer-induced bone disease (Fig. 5).

The UPR has been widely recognized for its role in oncogenesis, conferring a pro-survival and drug resistance benefit in many cancers.^173^ Due to the hypoxic, nutrient-deficient environment of the tumor niche, cancer cells often require activation of survival pathways like the UPR to combat these conditions.^6^ Therefore, it is no surprise that tumors exhibiting increased ER stress signaling correspond with poor prognosis and adverse treatment outcomes.^174–179^ This has fostered the development of UPR-targeting therapeutics for cancer, such as ERN1 inhibitors, EIF2AK3 inhibitors, along with agents targeting other UPR components, such as ERAD and chemical chaperones. Most of these inhibitors have only been evaluated in the realm of anti-tumor activity, without any studies on bone remodeling. This next section will summarize the efficacy of these therapies in bone-associated cancer, such as MM, OS, and bone-metastatic breast and prostate cancer, and review any preclinical studies which may inform the application of these therapies in the realm of bone health.

### EIF2AK3 inhibitors

EIF2AK3 plays multiple roles in oncogenesis such as promoting tumor angiogenesis and mediating hypoxia- and chemoresistance; therefore indicating potential for EIF2AK3-specific targeting for cancer treatment.^180–184^ Evaluation of first-generation EIF2AK3 inhibitor (GSK2656157) confirmed an anti-neoplastic benefit in various tumor xenograft models promoting the application of EIF2AK3 inhibitors for cancer treatment.^185,186^ Furthermore, investigation of a second-generation EIF2AK3 inhibitor (GSK2606414) revealed inhibitory effects on osteoclast precursor differentiation and autophagy.^153^ To elucidate any significant effects on bone remodeling, GSK2606414 was evaluated in ovariectomized (OVX) mice where it was found to restore bone density. Specifically, treatment was found to significantly increase trabecular bone density and reduce ACP5+ osteoclast numbers suggesting anti-resorptive effects in vivo.^153^ While these studies were not conducted in a setting of cancer-induced bone disease, the anti-tumor effects noted with the first generation EIF2AK3 inhibitor, suggests potential application in cancer-induced osteolytic bone disease.^185,186^

Translation of early generation EIF2AK3 inhibitors to the clinical setting has been difficult due to overt toxicity to pancreatic β-cells in humans, however next generation inhibitors with greater selectivity for EIF2AK3 are currently undergoing clinical trial development.^187,188^ While these inhibitors are not currently under investigation for cancer-associated bone disease, relevant clinical trials and potential bone-related effects are summarized in Table 1. Considering the effects of EIF2AK3 signaling on osteoclast/osteoblast differentiation, EIF2AK3 inhibition exhibits potential for dual therapeutic anti-neoplastic and bone-related benefits.

**Table 1:** Preclinical and clinical data summarizing the effects of promising UPR modulators in cancer

Drug	Mechanism of action	Preclinical data	Clinical data
HC-5404	Selective EIF2AK3 inhibitor	Anti-tumor activity in mouse models of renal cell carcinoma with evidence of synergy in combination with anti-angiogenic agents.^269^	Potential efficacy in heavily pretreated patients with solid tumors.^188^
NMS-03597812	ATP-competitive EIF2AK3 and GCN2 inhibitor	Anti-tumor activity in a mouse model of MM with evidence of synergy in combination with standard of care agents.^270^	Under investigation for treatment of RR acute myeloid leukemia (NCT06549790)
ORIN1001 (MKC-8866)	Selective ERN1 RNase inhibitor	Anti-tumor effects in mouse models of triple negative breast and prostate cancer with evidence of synergy when used in combination with current approved chemotherapeutics.^192,193,198^	Potential efficacy with single-agent use in a subset of patients with advanced solid tumors who exhibited disease stabilization or partial response^271^ Recently evaluated for treatment of RR breast cancer in combination with Abraxane (NCT03950570) but results are unavailable at this time
Sunitinib malate	Multi-targeted tyrosine kinase inhibitor, including ATP-competitive ERN1 kinase inhibitor	Anti-tumor activity in mouse models of OS.^199,200^	Approved for use in renal cell carcinoma, gastrointestinal stromal tumor, pancreatic neuroendocrine tumor
Toyocamycin	ERN1 ATP-dependent RNase inhibitor	Anti-tumor activity in a mouse model of MM.^201^	Lacked efficacy in patients with advanced solid tumors^202^
CB-5083, CB-5339	ATP-competitive VCP inhibitor	Anti-tumor activity in mouse models of MM and lung carcinoma.^272,273^	Under investigation for single agent and combination use with standard of care treatment for acute myeloid leukemia (NCT04372641, NCT04402541)
4-PBA	Histone deacetylase inhibitor that also acts as a chemical chaperone - binds exposed hydrophobic regions of misfolded proteins in the ER	Restored anti-tumor immunity in mice with ER stress induced tumor growth.^274^	Lacked efficacy as single agent and in combination for treatment of solid tumors and hematological malignancies.^275,276^
RAM2061	GGDPS inhibitor	Anti-tumor activity in mouse models of MM and ES.^223,225^ Reduced osteoclast number in CD-1 mice.^227^	None

*MM* multiple myeloma, *RR* relapsed/refractory, *OS* osteosarcoma, *EW* Ewing sarcoma, *GGDPS* geranylgeranyl diphosphate synthase

### ERN1 inhibitors

ERN1/XBP1 signaling is highly associated with cancer progression as elevated sXBP1 levels correlate with advanced disease stage and poor prognosis in MM, OS, breast and prostate cancer.^189–193^ Specifically in breast cancer, ERN1 signaling is activated as a result of hypoxia, with signaling inducing expression of hypoxia inducible factor 1 subunit alpha (HIF1A) and downstream targets, solute carrier family 2 member 1 (SLC2A1) and lactate dehydrogenase A (LDHA), incurring hypoxia-resistance and tumor growth.^191,194^ Anti-tumor effects in various cancer models have confirmed the anti-neoplastic potential of ERN1 inhibitors, with one inhibitor currently being evaluated in the clinical setting (ORIN1001) but bone-specific effects have yet to be evaluated (Table 1).^192,193,195–198^

Interestingly, the FDA approved, multi-targeted tyrosine kinase inhibitor, sunitinib malate, was found to also inhibit ERN1 autophosphorylation and RNase splicing activity. Evaluation of sunitinib treatment in a human xenograft OS mouse model indicated slowed tumor growth, reduced primary tumor vascularization, and inhibition of lung metastasis.^199^ Sunitinib single-agent treatment in a mouse model of bone metastatic breast cancer significantly reduced tumor growth and also decreased ACP5+ osteoclast numbers at the tumor-bone interface. However reduction in osteoclast number was not statistically significant.^200^ Toyocamycin, another ERN1 RNase inhibitor, has been shown to exhibit anti-MM activity in vivo due to inhibition of ERN1XBP1 signaling.^201^ However while phase I clinical trial (NSC-63701) investigation demonstrated an acceptable safety profile in humans, the lack of anti-tumor efficacy halted further development of toyocamycin.^202^ Considering the confirmed anti-neoplastic effect of ERN1 inhibitors and the role of ERN1 signaling in osteoclast differentiation (i.e., NFATC1 signaling), there is reason to believe that this class of drugs may have anti-resorptive benefits in the setting of osteolytic cancer-associated bone disease (Table 1).

### ER stress modulators

Valosin-containing protein (VCP) is an ERAD associated protein with known pathogenic mutations in bone disease like Paget’s and upregulation in cancer.^203,204^ VCP primarily functions by extracting misfolded polyubiquitinated proteins from the ER and delivering them to the proteasome for degradation. Therefore, VCP inhibition would increase misfolded protein load, inducing ER stress and potentially apoptosis. A VCP-specific inhibitor, VCP20, was found to prolong survival and improve bone destruction in a murine model of MM via disruption of NF-κB signaling in MM and osteoclast cells.^205^ VCP20 has yet to be evaluated in humans, however, other VCP inhibitors (CB-5083, CB-5339) have been clinically investigated over the years (Table 1). Unfortunately, due to issues with off-target toxicity the development of these compounds today has slowed.

Heat shock proteins (HSP), such as HSPA5, are molecular chaperones facilitating protein folding and UPR induction. Considering HSPs such as HSP90 are overexpressed and associated with poor prognosis in cancer, HSP90-selective targeting has become a popular area of interest with a total of 18 HSP90 inhibitors entering clinical trials.^206^ Unfortunately, due to dose-limiting toxicity and poor bioavailability, none have been approved for clinical use. Second-generation HSP90 inhibitor, ganetespib (STA-9090), exhibited potent anti-tumor effects and superior solubility in vivo suggesting greater potential for translation to the clinic. In vitro studies found HSP90 to mediate PTH activation of EIF2AK3 in osteoblast cells, with use of HSP90 inhibitor, geldanamycin, being found to decrease osteoblast EIF2AK3 protein levels in response to PTH treatment.^142,207^ Understanding PTH-induced EIF2AK3 signaling to mediate osteoblast TNFSF11 expression indicates a potential regulatory role for HSP90 in osteoblast-mediated TNFSF11 osteoclast activation. Understanding that ganetespib demonstrated safety and tolerability in phase I trials, supports further investigation into its potential application in cancer-induced bone disease.^206,208^

Sodium phenylbutyrate, or 4-phenylbutyrate (4-PBA), is a histone deacetylase inhibitor which also acts as a chemical chaperone, promoting protein folding and alleviation of ER stress. Evaluation of 4-PBA in a murine hindlimb suspension (HLS) model of disuse osteoporosis revealed bone-protective, and potentially anabolic, effects.^209^ This model utilized skeletally mature mice that underwent 3 weeks of HLS promoting generation of ROS in the bone, causing ER stress and significant loss of cortical and trabecular bone density.^209^ A subset of HLS mice receiving 4-PBA treatment for 21 days exhibited a significant degree of bone restoration, even more so than control mice which did not undergo HLS.^209^ The anti-osteoclastic effects of 4-PBA were attributed to downregulation of CTSK and ACP5 which were upregulated in HLS mice; however, the overall bone restorative effect is more likely attributed to alleviation of osteoblast ER stress.^209^ This is supported by enhanced mineral-to-matrix ratio, collagen maturity, and protein crosslinking following 4-PBA treatment, suggesting alleviation of ER stress in HLS osteoblasts, and therefore restoration of bone matrix protein processing and trafficking.

The osteoblast-restorative effect of 4-PBA has also been confirmed in other ER stress mediated pathologies. In a murine model of OI, osteoblasts were found to undergo ER stress-mediated apoptosis from inadequate collagen processing and secretion, but 4-PBA treatment alternatively promoted postnatal bone growth and reduced facture incidence.^210^ Furthermore, generation of osteoblast-specific ATG7 conditional knockout mice revealed reduced bone mass resulted from impaired osteoblast formation and matrix mineralization.^211^ Treatment with 4-PBA was found to ameliorate ER stress triggered in ATG7-deficient osteoblasts and restored function similarly to that seen in OI defective osteoblasts.^210,211^

4-PBA is currently approved for use in humans, albeit for the indication of urea cycle disorders. Multiple studies have failed to exhibit single-agent anti-tumor efficacy of 4-PBA in humans; however, it is still under investigation for treatment of solid tumors in combination with other chemotherapeutic agents (Table 1).

### Isoprenoid biosynthesis pathway inhibitors

Bisphosphonates, specifically the nitrogen-containing bisphosphonate zoledronic acid (ZA), are the frontline treatment option for patients with bone metastases or myeloma bone disease (MBD).^212^ NBPs exert their anti-osteoclastic effects by inhibiting enzyme farnesyl diphosphate synthase (FDPS) which mediates the formation of isoprenoid donors (farnesyl pyrophosphate (FPP), geranylgeranyl pyrophosphate (GGPP)) necessary for small GTPase post-translational modification (farnesylation, geranylgeranylation).^213,214^ Inhibition of Rho GTPase geranylgeranylation, is the recognized anti-osteoclastic mechanism behind NBPs, due to the role of Rho-regulated cytoskeletal processes in osteoclast differentiation and resorption.^213,215^ Aside from this direct anti-osteoclastic effect, ZA is thought to impart an anti-neoplastic benefit through direct and selective killing of tumor cells.^23^ In vitro studies confirmed anti-tumor properties such as ability to inhibit proliferation, adhesion, invasion, and induce cancer cell apoptosis, with clinical studies suggesting a potential survival benefit in MM, OS, and bone-metastatic breast or prostate cancer.^23,216–220^ Significant anti-tumoral effects of ZA in cancers associated with elevated UPR expression, suggests UPR-mediated apoptosis to potentially mediate ZA’s anti-neoplastic effects.^221^ While this could indicate application of ZA beyond the skeleton, potential is limited due to inability to reproduce anti-tumor effects in vivo. ZA has a 95% or greater affinity for hydroxyapatite (HAP; primary component of the bone matrix), therefore ZA distributes selectively to the bone, with remaining compound excreted unmodified by the kidneys.^212,222^ Thus, the lack of systemic distribution is the primary roadblock for application of NBPs for cancers outside of the bone.

The enzyme geranylgeranyl diphosphate synthase (GGDPS) directly follows FDPS in the isoprenoid biosynthesis pathway such that GGDPS inhibition prevents formation of only isoprenoid GGPP, without depleting FPP levels. Therefore, GGDPS inhibition presented a targeted strategy for selectively inhibiting protein geranylgeranylation without impacting farnesylation.^223^ GGDPS inhibitors (GGSIs) have demonstrated anti-neoplastic activity in malignancies characterized by aberrant protein production such as MM, by disrupting Rab-mediated protein trafficking, inducing ER stress, and UPR-mediated apoptosis.^223–225^ An α-methylated triazole bisphosphonate, RAM2061, was found to inhibit tumor growth and prolong survival in mouse models of MM and ES, and also, importantly, exhibits key drug-like features such as prolonged half-life, metabolic stability, and systemic distribution.^223,225^ Furthermore, RAM2061 in combination with proteasome inhibitor (PI) bortezomib, significantly reduced tumor growth in a MM xenograft model compared to either agent alone.^226^ While these studies confirmed anti-tumor effects, flank xenograft models did not allow for assessment of changes in bone remodeling, therefore, subsequent studies evaluated RAM2061 treatment on osteoclasts in vitro. RAM2061 exhibited anti-osteoclastic effects comparable to ZA, confirmed to result from disruption of Rho GTPase gernaylgeranylation.^227^ Evaluation of short course RAM2061 treatment in young CD-1 mice revealed no significant effects on bone morphology, volume, or serum levels of bone-formation or -turnover markers (CTX, P1NP, TRACP-5b).^227^ However, a decrease in ACP5+ osteoclasts was observed.^227^ HAP binding experiments determined RAM2061 to bind HAP with 60% affinity, which in conjunction with previously published distribution studies, raises the possibility that this agent could have both direct anti-tumor and anti-osteoclastic activity.

Novel thienopyrimidine-based GGSIs have also demonstrated anti-MM activity in mouse models of MM.^228–230^ While the investigators did not evaluate any bone parameters in these studies, they did evaluate the HAP affinity of their lead compound and found it bound HAP with affinity equivalent to ZA.^229^ While this suggests potential for potent bone-restoring effects, it likely limits the anti-tumor activity of this compound against cancer outside of the skeleton.

### Proteasome inhibitors

PIs are one of the most well-studied classes of UPR modulators. The ubiquitin-proteasome system (UPS) mediates the degradation of more than 80% of cellular proteins.^231^ Therefore, UPS inhibition significantly increases misfolded protein load, triggering prolonged UPR signaling, and subsequent apoptosis.^232^ Bortezomib was the first-in-class, boronate-based reversible PI to receive FDA approval after it was found to improve progression-free survival and overall survival of patients with MM.^233,234^ While emerging as one of the most effective treatments for MM, it was quickly recognized for its additional bone-restorative benefits.^235–237^ At the cellular level, bortezomib was found to promote bone formation through activation of ERN1/XBP1 signaling and ATF4 expression in osteoblasts, promoting differentiation and maturation. This pro-osteoblastic effect was confirmed in various clinical trials where bortezomib was found to elevate serum levels of bone formation markers such as bone ALP (bALP) and BGLAP and decreased levels of osteoblast inhibitor DKK1.^238–241^ Importantly, bone anabolic effects were specifically attributed to PIs, with inclusion or exclusion of bortezomib from combination treatment regimens differentially impacting bone disease.^242,243^ Bortezomib was also found to reduce serum levels of bone resorption markers (CTX, TRACP-5b) and osteoclast activator, soluble TNFSF11 (sTNFSF11), suggesting an anti-resorptive benefit.^244,245^ This was confirmed with bortezomib found to inhibit osteoclast differentiation through disruption of UPS mediated degradation of NF-κB inhibitor, IκB, and inhibition of MAPK14 (also known as p38) signaling during osteoclast differnetaition.^246,247^ This supported further investigation into the bone-specific effects of bortezomib, including an observational study (NCT01026701) evaluating the effects of intravenous bortezomib on osteoblast activity in patients with MM. While the study has been completed, results are not available at this time.

Due to development of drug resistance and dose-limiting side effects (e.g., peripheral neuropathy), second-generation PIs were developed. Carfilzomib, an epoxyketone-based irreversible PI, demonstrated safety, tolerability, and anti-tumor activity in humans leading to fast-track FDA approval for single agent use in patients with RR MM.^248–252^ The bone anabolic effects of carfilzomib were demonstrated in vivo with non-tumor bearing mice and mice with disseminated MM exhibiting increased trabecular bone volume, decreased bone resorption, and enhanced bone formation.^253^ Bone-forming effects were confirmed to result from ERN1/XBP1 signaling in osteoblasts as expected, but promotion of osteogenesis was attributed primarily to activation of WNT-dependent β-catenin signaling.^254^ Interestingly, carfilzomib was also found to exhibit anti-osteoclastic effects unseen with bortezomib, such as disrupting TNFSF11-induced NF-κB signaling and PTH-induced osteoclastogenesis.^255^ The specific bone metabolic effects of carfilzomib were confirmed in a phase II study which found single agent use in patients with RR MM to decrease serum markers of bone resorption (CTX, TRACP-5b).^256^ Significant changes in bone formation markers (P1NP, OCN) were generally not observed, except in patients who achieved hematological partial response or better.^256^ Similarly, a prospective study evaluating the impact of carfilzomib with dexamethasone on bone metabolism in RR MM found significantly decreased serum levels of bone resorption markers, CTX and TRACP-5b.^254^ Importantly, an increase in serum levels of bone formation markers P1NP, OCN, bALP was also noted.^254^ Whether this is due to a direct bone-anabolic effect or indirect due to reduced MM disease burden requires clarification.

Ixazomib was the first oral PI to receive FDA approval following confirmation that the addition of ixazomib to lenalidomide and dexamethasone significantly increased PFS compared to lenalidomide and dexamethasone alone.^257^ Ixazomib is similar to bortezomib in that it is a boronate-based, reversible PI. However, evaluation of ixazomib and bortezomib in vivo revealed ixazomib to be superior in reducing tumor burden and preventing bone loss in MM.^258^ Ixazomib bone anabolic effects were confirmed to result from ERN1/XBP1 signaling activation and β-catenin signaling in osteoblasts, with concurrent inhibition of NF-κB signaling and osteoclast formation.^258^ An open label phase II study (NCT04028115) found 3-month long treatment to impart a net bone-anabolic effect in patients with MM with evidence of decreased CTX and TRACP-5b serum levels, however P1NP and bALP bone formation markers seemed unaffected.^259^ Evaluation of ixazomib treatment in other indications such as OS showed in vivo treatment enhanced overall survival by slowing primary tumor metastasis and inhibiting growth of pulmonary and abdominal metastases; while bortezomib only inhibited the growth of pulmonary metastases. This study revealed the potential application of ixazomib treatment in OS and suggested that ixazomib has greater ability to penetrate tumors due to superior pharmacokinetic and pharmacodynamic properties.^258,260,261^

Due to issues of off-target toxicities and drug resistance with early generation PIs, third-generation PIs were developed. Oprozomib, an orally administered derivative of carfilzomib, exhibited significantly greater anti-osteoclastic activity in vitro compared to bortezomib, and conferred an anti-tumor benefit in MM in vivo.^253^ Furthermore, oprozomib dose-dependently increased ALP activity and mineralization of osteoblasts in vitro which was a direct result of ERN1 activation, due to lack of mineralization with oprozomib treatment in ERN1 knockout cells.^253^ Phase I studies (NCT01129349, NCT01365559) revealed tolerability and efficacy in patients with solid tumors and hematological malignancies, however gastrointestinal (GI) related toxicities were common suggesting the need for a formulation change.^262^ GI toxicity from oprozomib was ameliorated when used in combination with dexamethasone (NCT01832727) supporting the continued investigation of two different oprozomib formulations in combination with dexamethasone and pomalidomide for RR MM (NCT02939183).^263,264^

Marizomib is unique from other PIs in that it is a naturally occurring compound derived from marine bacterium. Mechanistically, it irreversibly binds and inhibits all three active sites on the proteasome, suggesting prolonged pharmacodynamic activity. Marizomib was found to be superior in its ability to inhibit NF-κB signaling and inhibiting osteoclastogenesis compared to bortezomib.^265^ Furthermore, it was found to induce apoptosis in bortezomib-resistant MM cells due to a slightly different apoptotic mechanism leading to inhibition of tumor growth and prolonged survival in MM in vivo.^266^ Phase I and II (NCT00396864, NCT00629473, NCT00461045) investigation in patients with solid tumors or hematological malignancies revealed lack of severe peripheral neuropathy or hematological toxicity seen with first-generation PIs, and demonstrated efficacy in patients with bortezomib-resistance.^267,268^ Application has continued to be pursued in combination use with pomalidomide and dexamethasone in relapsed/refractory MM (NCT02103335, NCT05050305), and due to its unique ability to cross the blood-brain-barrier, marizomib is undergoing investigation in central nervous system (CNS) involved MM (NCT05050305) and glioblastoma (NCT02330562, NCT03345095).

## Conclusions

The UPR is tightly integrated with bone cell differentiation and function and plays a salient role in oncogenesis. Bone is the third most common site of metastasis and localization of cancer cells to the medullar cavity results in disruption of normal homeostatic processes, including bone remodeling, leading to secondary bone disease. Understanding the connection between the UPR and cancer-associated bone disease presents a unique opportunity to dually target cancer cells and promote restoration of bone remodeling. While the FDA-approved agents sunitinib malate and 4-PBA have UPR modulatory activities, the effects on bone parameters in humans have not been reported. Development of next generation EIF2AK3 inhibitors or ERN1 inhibitors may yield selective agents which can directly modulate the UPR in cancer-associated bone disease. Considering that tumor cell metabolism varies based on primary vs metastatic sites, future studies should aim to clarify whether UPR modulation and targeting is altered by tumor location. PIs are widely used for treatment of MM and MBD, and PI treatment was found to incur significant bone anabolic benefits. Further clarification on whether PIs abrogate bone disease in the setting of other primary-bone or metastatic-bone cancers remains to be determined. While current agents for cancer-induced bone disease such as ZA and denosumab prevent further incidence of SREs, the magnitude of effect on the tumor itself has yet to be clarified. Emerging therapeutic classes such as the GGSIs hold promise due to their anti-osteoclastic and anti-tumor effects, but further studies are required to determine the extent to which these agents can abrogate cancer-induced bone disease in vivo. Ultimately, the successful application of UPR modulators for cancer-induced bone disease will be dependent on agents having the requisite pharmacokinetic and biodistribution properties to enable sufficient bone exposure as well as specific on-target effects on bone and tumor while minimizing off-target toxicities.
